# Low-dose radiation ameliorates doxorubicin-induced renal injury via reducing oxidative stress and protecting mitochondrial function

**DOI:** 10.1371/journal.pone.0313649

**Published:** 2025-02-11

**Authors:** Mengmeng Chen, Kang He, Kai Wang, Yibo Cai, Zhaohui Ying, Xueting Li, Yating Liu, Liting Xiang, Pingping Yang, Hongjuan Wu, Jian Jiang

**Affiliations:** 1 Department of Rehabilitation, School of Nursing, Jilin University, Changchun, China; 2 Department of Nursing, Zhejiang Cancer Hospital, Hangzhou, China; King Saud University, SAUDI ARABIA

## Abstract

Doxorubicin (DOX) is a well-established chemotherapy drug, but its clinical application is restricted due to significant tissue toxicity, of which nephrotoxicity is a serious adverse reaction. Low-dose radiation (LDR) exerts effects through stimulating diverse cell and molecular mechanisms, which has been shown to have anti-inflammatory and alter immune adaptation effects. This study aims to investigate how LDR protects against DOX-induced nephrotoxicity and to explore the underlying mechanism involved. Sixty mice were randomly divided into control (CTR), LDR, DOX, and combination (COM) group. Nephrotoxicity was induced by injecting a single dose of DOX (7.5 mg/kg) in mice abdominal cavity, and LDR was performed 72 h before DOX treatment. Histological analysis, immunohistochemical analysis, immunofluorescence analysis and western-blotting were used to detect the related indicators. Research data was showed as mean ±SD and tested by One-way ANOVA. The results showed that compared with DOX group, the contents of serum UREA, UA, and the expression level of Bax and caspase 9 were significantly reduced in COM group (*P<*0.05). Western-blotting and immunohistochemical analysis showed that the expression level of MDA and Nrf2 in COM group were significantly lower than that in DOX group (*P<*0.05). In addition, the activity of complex Ⅰ, ATP, NDUFA1 and CYC1 were enhanced in COM group compared with DOX group (*P<*0.05). All the results suggested that LDR pretreatment prevented excessive accumulation of peroxides, restored antioxidants activity (SOD, GSH, CAT), activated Nrf2/HO-1/NQO1 signaling pathway, attenuated damage to the mitochondrial respiratory chain, and protected kidney cells from DOX attack. This study demonstrated that LDR could effectively and safely inhibit the progression of DOX-induced nephrotoxicity. Future studies should further investigate the mechanism of LDR protecting tissues from DOX-induced damage and find an optimal radiation dose for humans.

## Introduction

Doxorubicin (DOX) is a classic anti-tumor medicine used to treat breast cancer, acute leukemia, malignant lymphoma and other tumors [1, 2]. The appreciated anti-tumor mechanism of DOX is to promote cell death or apoptosis by disrupting cell division or inhibiting the synthesis of RNA and DNA [3]. However, its severe dose-dependent side effects during the antitumor process, especially nephrotoxicity and cardiotoxicity caused by the accumulation of reactive oxygen species (ROS), limit its clinical application [4–7]. Despite many attempts are performed to reduce the side effects of DOX, such as chemical modification, nanomaterial loading [8–11], there is still no strategy that could actually protect the organs and tissues from DOX without compromising the anti-tumor effect. Therefore, finding suitable strategy is of great importance for current clinical cancer treatment.

Low-dose radiation (LDR), defined as a radiation dose of 100 mGy or less [12], has been shown to have anti-inflammatory effects and alter immune adaptation [13–15]. Inconsistent with high-dose radiation that rapidly kills cells, whether normal or tumor cells, LDR could activate certain protection mechanisms prior to radiotherapy and chemotherapy, thereby improving cellular fitness and lessening damage [16, 17]. A previous study have shown that LDR could stimulate the growth of normal cells in *vitro*, but not leukemia or solid tumor cells [18]. In addition, LDR preprocessing could reduce cancer risk by stimulating anti-cancer immunity, and alleviate the liver damage induced by cyclophosphamide [19]. These effects were attributed to the enhanced activity of antioxidants [20] and DNA repair enzymes by milder pre-stimulation of LDR [21, 22]. At present, the protective effect of LDR has attracted widespread attention, but its protective mechanism against DOX-induced renal injury remains unclear.

## Materials and methods

### Chemical reagents

Doxorubicin hydrochloride was purchased form Yuanye Bio-Technology Co., Ltd., and used as received. Antibodies were mainly purchased from the company of Proteintech Group, Inc. and Affinity Biosciences, Ltd. Terminal deoxynucleotidyl transferase biotin-d UTP nick end labeling (TUNEL) fluorescent assay kit was purchased from Roche Biotech, Inc. The tissue mitochondrial extraction kit and ROS detection kit were purchased from Beyotime Biotechnology, Inc. Other biochemical kits were purchased from two Chinese biotechnology companies (Jiancheng Bioengineering Institute and Solarbio Science & Technology, Ltd.). All antibodies and kits were used in strict accordance with the instruction manuals. Specific information about the chemical reagents were provided in **S1 Table in S2 File**.

### Animals

In this study, BALB/c female mice were randomly divided into 4 groups: control (CTR) group, LDR, DOX, and combination (COM) group. The 7.5mg/kg dosing DOX was used to construct the kidney injury model [23, 24] for exploring the effect of LDR on mice kidney. Sixty 6-week-old BALB/c female mice were obtained from Beijing Unilever Laboratory Animal Co., LTD. The in vivo experiments were conducted using protocols and conditions approved by the Experimental Animal Ethics Committee of Jilin University (Ethics number: 202073). Animal experiments were complied with The ARRIVE guidelines 2.0 and the National Institutes of Health Guidelines for the Care and Use of Laboratory Animals. Female male BALB/c mice were reared in a group of five animals per cage at a temperature of 22±1°C and a 12 h light/dark cycle with access to food and water ad libitum.

**Fig 1:**
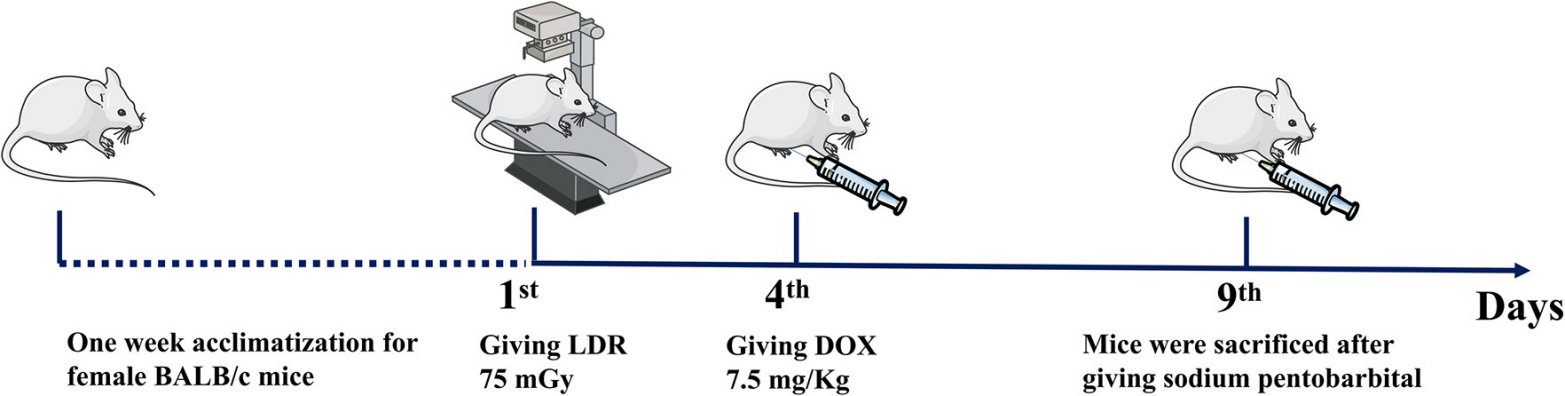
The timeline of animal experiments.

After one week acclimatization, mice were randomly divided into 4 groups (*n* = 15): ① CTR: 0 mGy radiation was performed at the 1^st^ day, and saline was injected intraperitoneally at the 4^th^ day; ② LDR: 75 mGy radiation was performed on the whole body at the 1^st^ day using Philips deep X-ray machine (XSZ-220/20) at dose rate of 0.0133 Gy/min for a total exposure time of 5.64 minutes, and saline was injected intraperitoneally at the 4^th^ day; ③ DOX: 0 mGy radiation was performed at the 1^st^ day, and 7.5 mg/kg doxorubicin was injected intraperitoneally at the 4^th^ day; ④ COM: 75 mGy radiation was performed at the 1^st^ day, and 7.5 mg/kg doxorubicin was injected intraperitoneally at the 4^th^ day. Body weight of the mice, as an outcome variable, was measured on the 1^st^ and 9^th^ days in the experiment. Following blood collection under three anesthesia treatments on 9^th^ day, the mice were euthanized by cervical dislocation under 1% sodium pentobarbital anesthesia (**Fig 1**). The kidney samples were collected, and stored at −80°C or fixed in 10% formalin.

### Serum biochemical examination

Blood was collected and centrifuged at 3000 g for 10 min at 4°C, and then the serum was extracted and stored at -80°C. The level of CREA, UREA, UA and CO2-CP in serum were measured following instructions of the assay kits.

### Histological analysis

Kidney specimens were fixed in 4% paraformaldehyde, embedded in paraffin, and cut into 4 μm thick slides. The histopathological alteration was examined under light microscope (Olympus IX71, Olympus Corporation, Tokyo, Japan) after stained with hematoxylin and eosin (H&E) and periodic acid-schiff (PAS). A minimum of 10 fields for each kidney slide were examined and scored for pathological injury. A score from 0 to 4 was given for pathological assessment: 0, normal histology; 1, mild injury, 5% to 25% of tubules showed pathological damage; 2, moderate injury, 25% to 50% of tubules showed pathological damage; 3, severe injury, 50% to 75% showed pathological damage; and 4, almost all tubules in field of view were damaged. The average histological score for each sample was calculated.

### TUNEL assay

The cell apoptosis rate was measured by TdT-mediated dUTP Nick-End Labeling (TUNEL) in situ cell death detection kit according to the manufacturer’s instructions. Detection of the apoptotic cells showing green fluorescence was performed by fluorescence microscopy.

### Immunohistochemical (IHC) analysis

For immunohistochemical staining, the endogenous peroxidase activity was blocked by 3% hydrogen peroxide solution. Epitope retrieval was performed by 10 mM sodium citrate buffer (pH 6.0) and heating twice in microwave oven. Non-specific binding was prevented by goat serum (1:10 in PBS). After incubation with the primary antibody (1:100 in PBS), the slides were incubated with biotinylated goat anti-rabbit IgG, followed by incubation with 1:200 streptavidin-biotin-peroxidase complex. Reactive products were visualized with 3,3′-diaminobenzidene (DAB) as the chromogen, and the slides were counterstained with hematoxylin. The brown staining of renal medulla was scored using Image-Pro Plus 6.0.

### Immunofluorescence analysis

To visualize the expression and localization of Nrf-2 in cultured renal cells, the slides were incubated with a rabbit Nrf-2 antibody as a primary antibody at 4°C overnight. Then slides were incubated in Alexa Fluor® 488 dye (1:400, Invitrogen) for 60 min. Finally, slides were visualized using Carl Zeiss LSM 5 PASCAL laser scanning confocal microscopy.

### Measurement of ROS

ROS fluorescent probe was used according to the manufacturer’s protocol. The whole kidney was grinded with cool PBS at 4°C, filtrated with gauze and centrifuged at 1200 rpm for 5 min to obtain the single kidney cells. After washed twice with PBS, the kidney cells were resuspended with serum-free dulbecco’s modified eagle medium (DMEM) with 2’,7’-Dichlorodihydrofluorescein diacetate (DCFH-DA) and cultured at 37°C for 30 minutes. Then, the cells were washed with PBS three times and resuspended in serum-free DMEM. Finally, the cells were imaged with fluorescence microscope (Optitec-YG-100). The fluorescent intensity was quantified by Image-J.

### The level of oxidative stress markers

10% kidney tissue homogenate was prepared to detect the activity of SOD, CAT and GSH, and the concentration of MDA. All the oxidative stress associated markers were measured by assay kits.

### The mitochondrion complexes and ATP content analysis

The concentration of ATP and the activity of mitochondrion complexes, including complex Ⅰ, complex Ⅱ and complex Ⅲ, were detected by assay kits.

### Western blotting (WB) assays

The mitochondria of kidney tissues were collected by the tissue mitochondrial extraction kit. The total protein samples or mitochondrion protein samples from kidney were extracted with cold RIPA lysis buffer with proteinase inhibitors, separated in 12% SDS-PAGE gels electrophoretically and transferred onto Polyvinylidene Fluoride membranes (Millipore, USA). The non-specific binding sites of the membranes were blocked with 5% dried skim milk at 37°C for 1 h. Then the membranes were incubated with primary antibodies at 4°C, followed by incubation with horseradish peroxidase tagged secondary antibody (Bioss, China) at room temperature for 2 h. Finally, the protein level was detected with an electrochemiluminescence plus kit (Affinity, China), and the densities of the specific bands were quantified with an imaging densitometer (Clinx Science Instruments Company, China) [25]. The GAPDH and β-tubulin were used as the internal references of total protein, and COX4 was used as the internal reference in mitochondria protein.

### Statistical analysis

All results were analyzed using SPSS ver. 26 (IBM, Inc) and GraphPad Prism 5 (GraphPad Software, Inc). Data was showed as mean ± standard deviation (SD). One-way ANOVA test followed by the Multiple Comparisons Tukey’s test was performed to evaluate differences between the groups, and *P*<0.05 was considered as statistical significance.

## Results

### LDR alleviated renal damage induced by DOX

**Fig 2:**
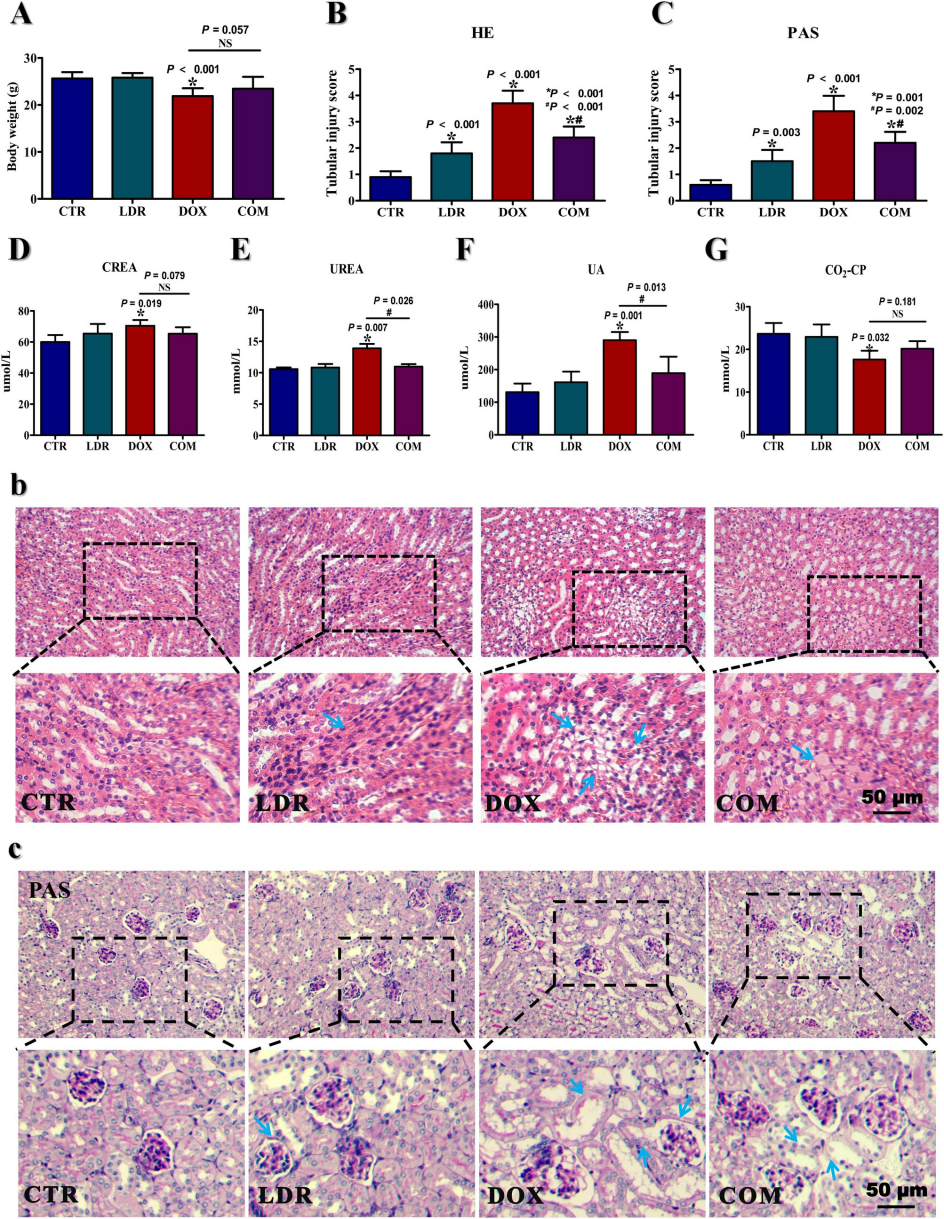
LDR alleviated kidney toxicity induced by DOX. Serum from 10 mice in each group was used to detect biochemical indicators of renal function, and kidney sections from 3 mice in each group were used for H&E and PAS staining. A) The concentration of CREA in serum. B) The concentration of UREA in serum. C)The concentration of UA in serum. D)The concentration of CO2-CP in serum. E) H&E staining of kidney tissues. F) PAS staining of kidney tissues. The blue arrows in figure E and F pointed to the areas of injury. “*” represents the significance difference between treatment groups and CTR group (*P*<0.05). “#” represents the significance difference between COM group and DOX group (*P*<0.05). Abbreviation: CTR, control group; LDR, low-dose radiation group; DOX, doxorubicin group; COM, combination group; UREA, urea nitrogen; CREA, creatinine; UA, uric acid; CO_2_-CP, carbon dioxide combining power.

After intervention, the weight of the mice was measured, and the result showed that compared with CTR group, the mice in DOX group lost significant weight (*P*<0.001), while there was no significant change in COM group (**Fig 2A**). To investigate the morphological changes, kidney slides were stained with H&E and PAS. Compared with CTR group, slight injury was seen locally in LDR group, and the cytoplasmic vacuolization of renal tubular epithelial cells and focal necrosis of kidney tissue were observed in DOX group. The tubular injury scores were statistically significant (*P*<0.001). Compared with DOX group, the renal tissue necrosis and the vacuolization of the cytoplasm alleviated obviously in COM group (*P*<0.001), as shown in **Fig 2B** and **2b**. The results of PAS staining (**Fig 2C**) showed that compared with the CTR group, the kidney of DOX group showed obvious features of renal tubule injury, including tubular dilation, brush boundary loss, cytoplasmic vacuole formation, cast formation, and inflammatory cell infiltration, the tubular injury scores were statistically significant (*P*<0.001). Strikingly, these histological lesions were significantly weakened in mice pre-protected with LDR. There was statistical significance in tubular injury scores between DOX and COM group, and the *P* value was 0.002 (**Fig 2c**).

In order to clarify the effect of interventions on renal function, the concentration of serum CREA, UREA, UA and CO_2_-CP in mice were tested (**Fig 2D–2G**). The results showed that the levels of CREA, UREA and UA were significantly increased in DOX group compared with CTR group (*P*<0.05), but no obvious difference was observed between the LDR, COM and CTR group. The concentration of UREA (*P* = 0.0026) and UA (*P* = 0.013) in COM group were significantly less than that in DOX group. Meanwhile, the CO_2_-CP was significantly lower in DOX group compared to CTR group (*P* = 0.032).

### LDR decreased DOX-induced apoptosis

**Fig 3:**
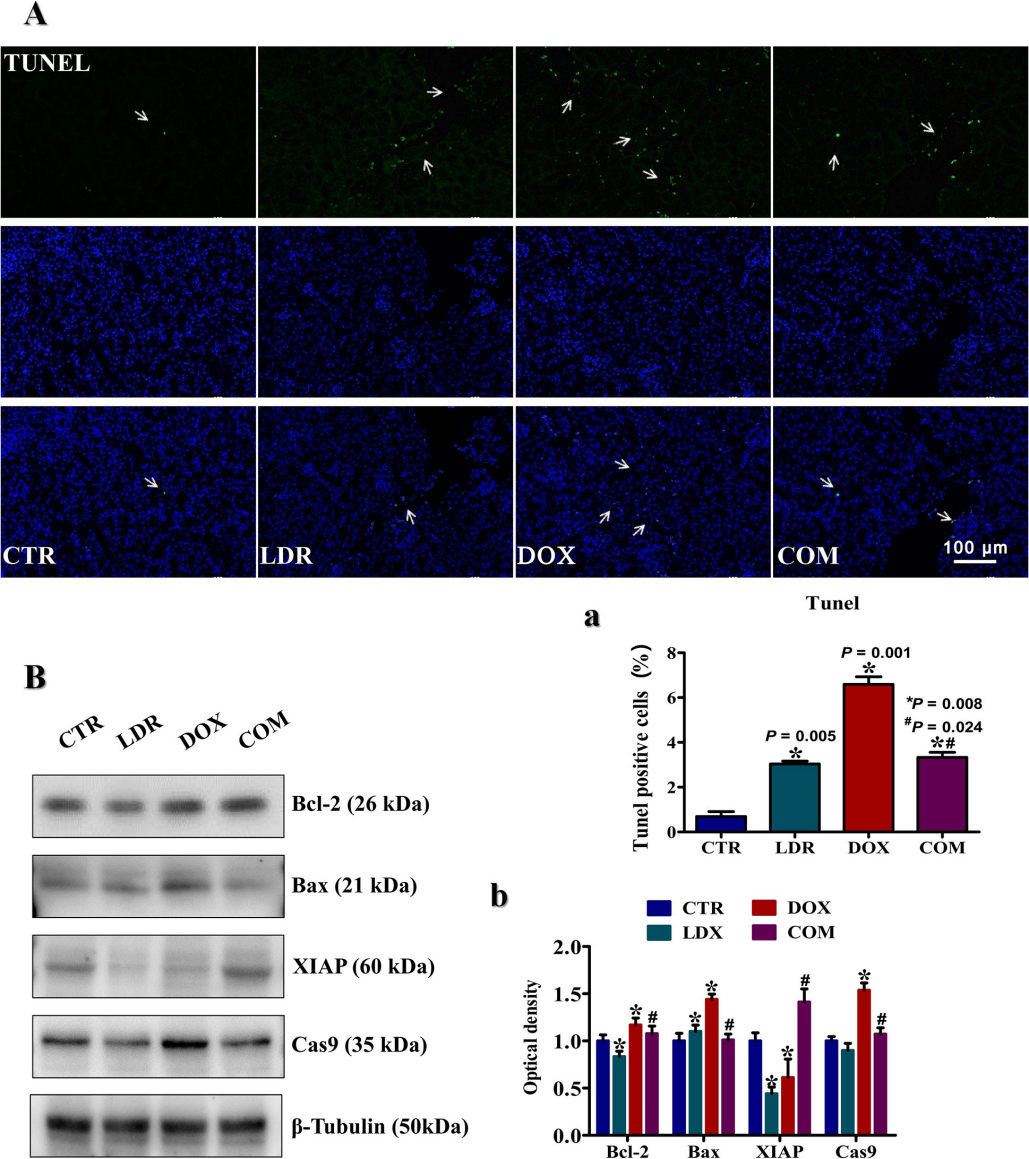
Cell apoptosis in kidney with different treatments. A) TUNEL stained kidney sections with different treatments (×200). B) Bcl-2, Bax, XIAP, and Caspase9 level in kidney tissues. All data were obtained from 3 mice per group. The white arrows in figure A pointed to the areas of high fluorescence intensity. “*” represents the significance difference between treatment group and CTR group (*P*<0.05). “#” represents the significance difference between COM group and DOX group (*P*<0.05). Abbreviation: CTR, control group; LDR, low-dose radiation group; DOX, doxorubicin group; COM, combination group; TUNEL, terminal deoxynucleotidyl transferase biotin-d UTP nick end labeling; Cas9, caspase9.

In order to evaluate the effect of LDR pretreatment on renal cell apoptosis under DOX treatment, TUNEL staining was performed on renal tissue sections. The green fluorescence indicated apoptosis, as shown in **Fig 3A**. Quantitative analysis (**Fig 3A)** for the apoptotic index showed that the fluorescence intensity increased in all intervention groups compared to CTR group, especially in DOX group (*P* = 0.001). However, the intensity of green fluorescence in COM group was significantly lower than that in DOX group (*P*<0.05).

Then, the protein expression of apoptosis-related pathway was examined by western blot **(Fig 3B** and **3b**). The results showed that the ratio of Bcl-2/Bax was 0.82 in CTR group, and the level of Cas9 was similar with those in CTR group. In DOX group, the ratio of Bcl-2/Bax was 0.74, the XIAP expression was significantly down-regulated, and the level of Cas9 was significantly increased compared with CTR group (*P*<0.05). In COM group, the ratio of Bcl-2/Bax was 1.07, the expression of XIAP was significantly elevated compared to DOX group, and the level of Cas9 was significantly decreased than that in DOX group (*P*<0.05).

### LDR attenuated DOX- induced oxidative stress

**Fig 4:**
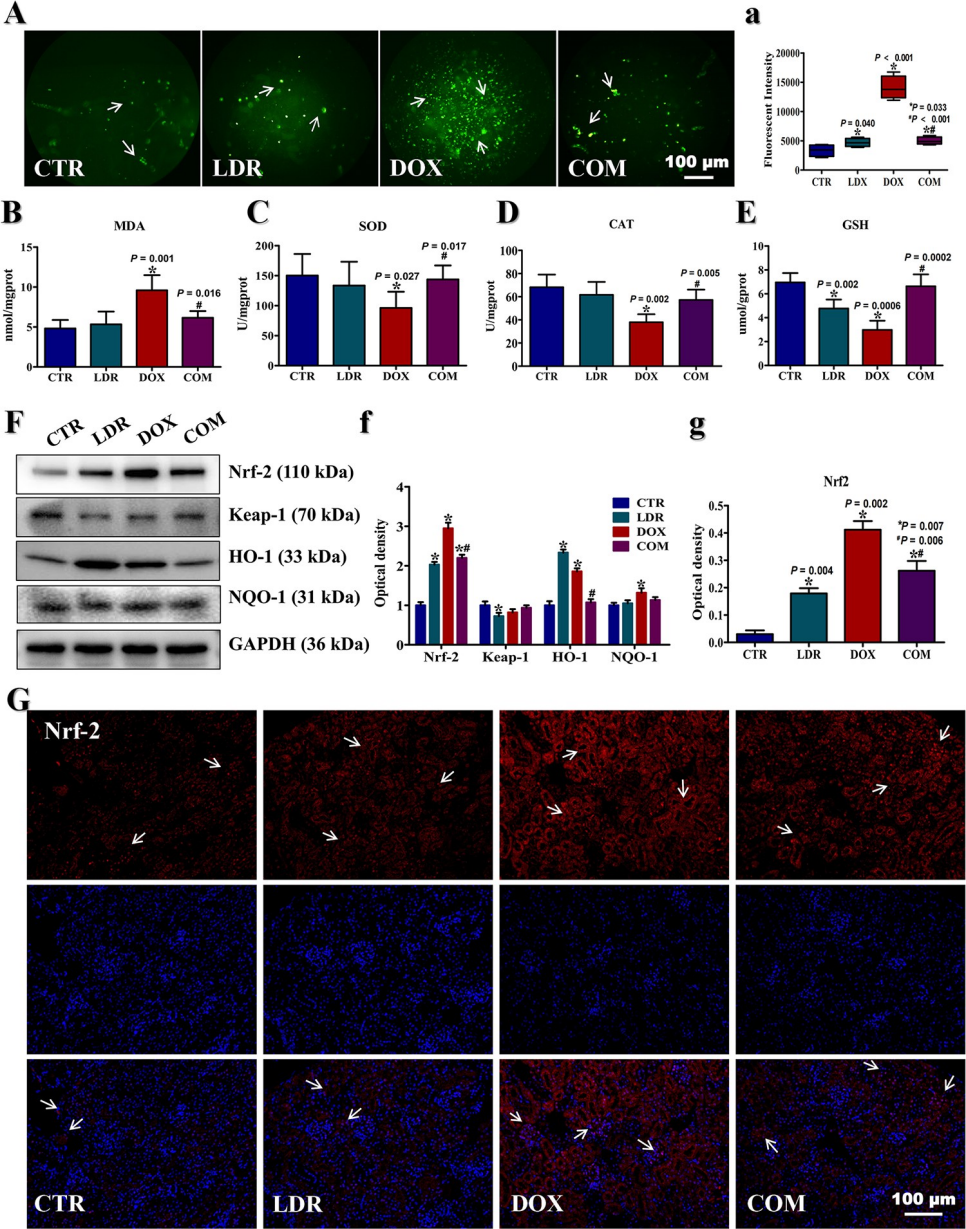
Oxidative stress in kidney with different treatments. A) ROS staining kidney cells with different treatments (×200). B) MDA content. C) SOD activity. D) CAT activity. E) GSH activity. F) The level of Nrf-2, Keap-1, HO-1 and NQO-1 in the kidney tissues. G) Immunofluorescence staining of Nrf-2 in kidney tissues (×200). All data were obtained from 3 mice per group. The white arrows in figure A and G pointed to the areas of high fluorescence intensity. “*” represents the significance difference between treatment group and CTR group (*P*<0.05). “#” represents the significance difference between COM group and DOX group (*P*<0.05). Abbreviation: CTR, control group; LDR, low-dose radiation group; DOX, doxorubicin group; COM, combination group; ROS, reactive oxygen species; MDA, malondialdehyde; SOD, superoxide dismutase; CAT, catalase; GSH, glutathione.

To further explore the protective mechanism of LDR on renal injury, oxidative distress injury in kidney cell was detected. The results showed that intracellular DCFH was oxidized by oxidant to DCF with green fluorescence, as shown in **Fig 4A**. Quantitative analysis (**Fig 4A**) suggested that the highest degree of DCFH was oxidized in DOX group (DOX vs CTR, *P*<0.001). Although the fluorescence intensity of LDR group was also increased (LDR vs CTR, *P* = 0.04), it was weaker than that in DOX group. It was noteworthy that the green fluorescence in COM group was significantly less than that in DOX group, and the fluorescent intensity score was statistically significant (*P*<0.001).

Then, the contents of MDA, SOD, CAT and GSH in renal tissues were assessed (**Fig 4B–4E**). The results showed that the level of MDA in DOX group was increased compared with CTR group, and the difference was statistically significant (*P* = 0.001). At the same time, the activities of SOD, CAT and GSH in DOX group were significantly lower than those in CTR group (*P*<0.05). Compared with DOX group, the content of MDA was significantly lower in COM group (*P* = 0.018), while the oxidative stress enzyme activities were all elevated to different degrees (*P*<0.05). In addition, there was a trend of elevated MDA content, and significantly lower GSH activity in LDR group compared with CTR group (*P* = 0.002).

To further explore the oxidative stress mechanism, this study investigated the expression of Nrf-2/HO-1/NQO1 signaling pathway, as shown in **Fig 4F** and **4f**. Western blot results showed that the level of Nrf-2 in LDR, DOX and COM groups were significantly up-regulated than CTR group (*P*<0.05). While the expression level of Keap1 was almost identical, the Nrf-2 level in COM group was significantly lower than DOX group (*P*<0.05). The quantification of protein blots showed that the expression of HO-1 and NQO-1 were significantly upregulated in DOX group than in CTR group (*P*<0.05). Compared with the DOX group, the COM group showed a significant decrease in HO-1 expression (*P*<0.05). The immunofluorescence staining assay (**Fig 4G, 4g**) showed that Nrf-2 in LDR, DOX and COM groups were significantly up-regulated both in the nucleus and in the cytoplasm compared with CTR group (*P*<0.05).

**Fig 5:**
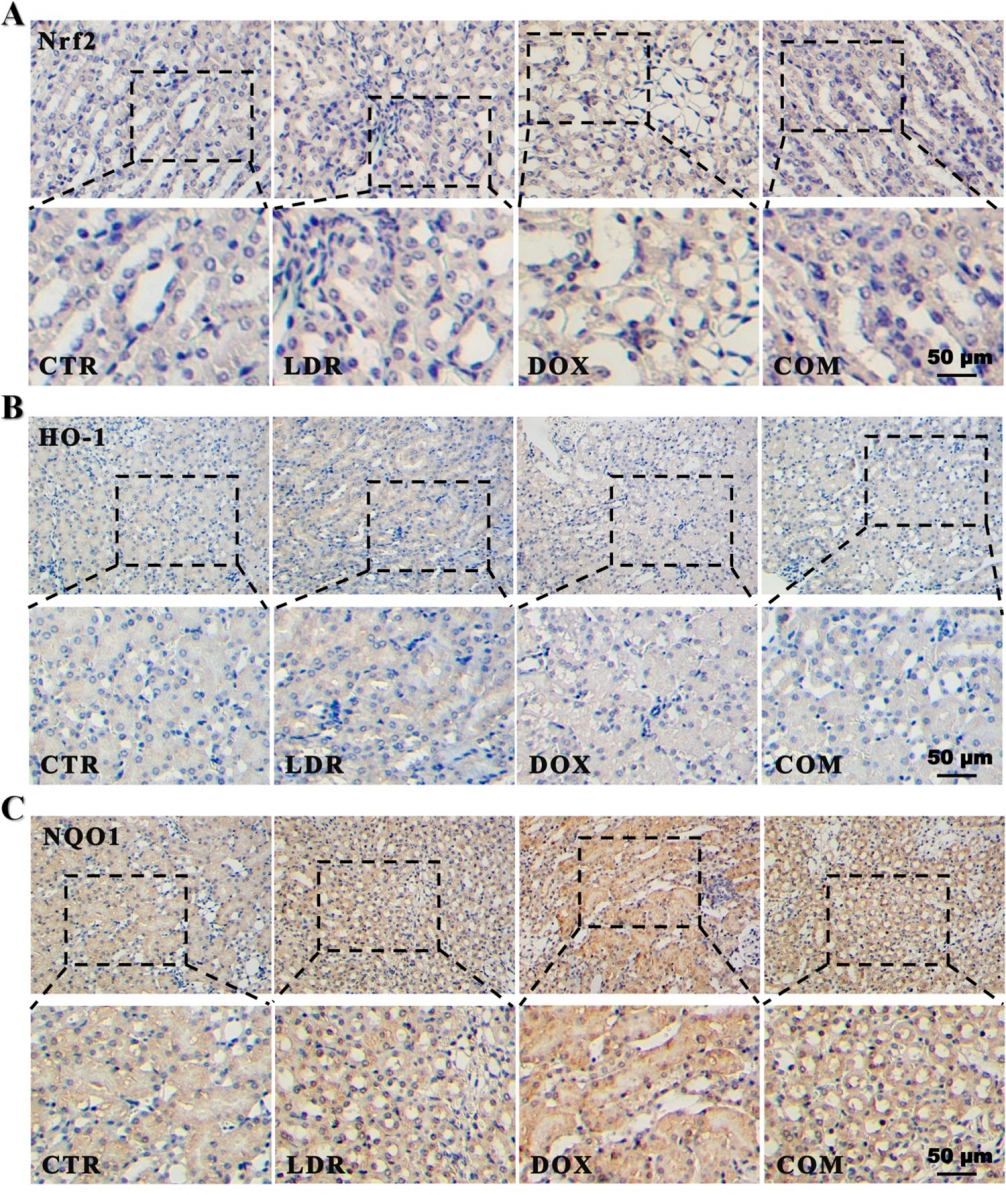
Immunohistochemical analysis. A) IHC staining of Nrf-2, B) HO-1, and C) NQO1 in kidney tissues. All data were obtained from 3 mice per group. Abbreviation: CTR, control group; LDR, low-dose radiation group; DOX, doxorubicin group; COM, combination group.

In addition, the IHC assay showed that the trend of Nrf-2, HO-1 and NQO1 expression in the four groups was consistent with the above experimental results (**Fig 5A–5C**).

### LDR protected mitochondrion from DOX toxicity

**Fig 6:**
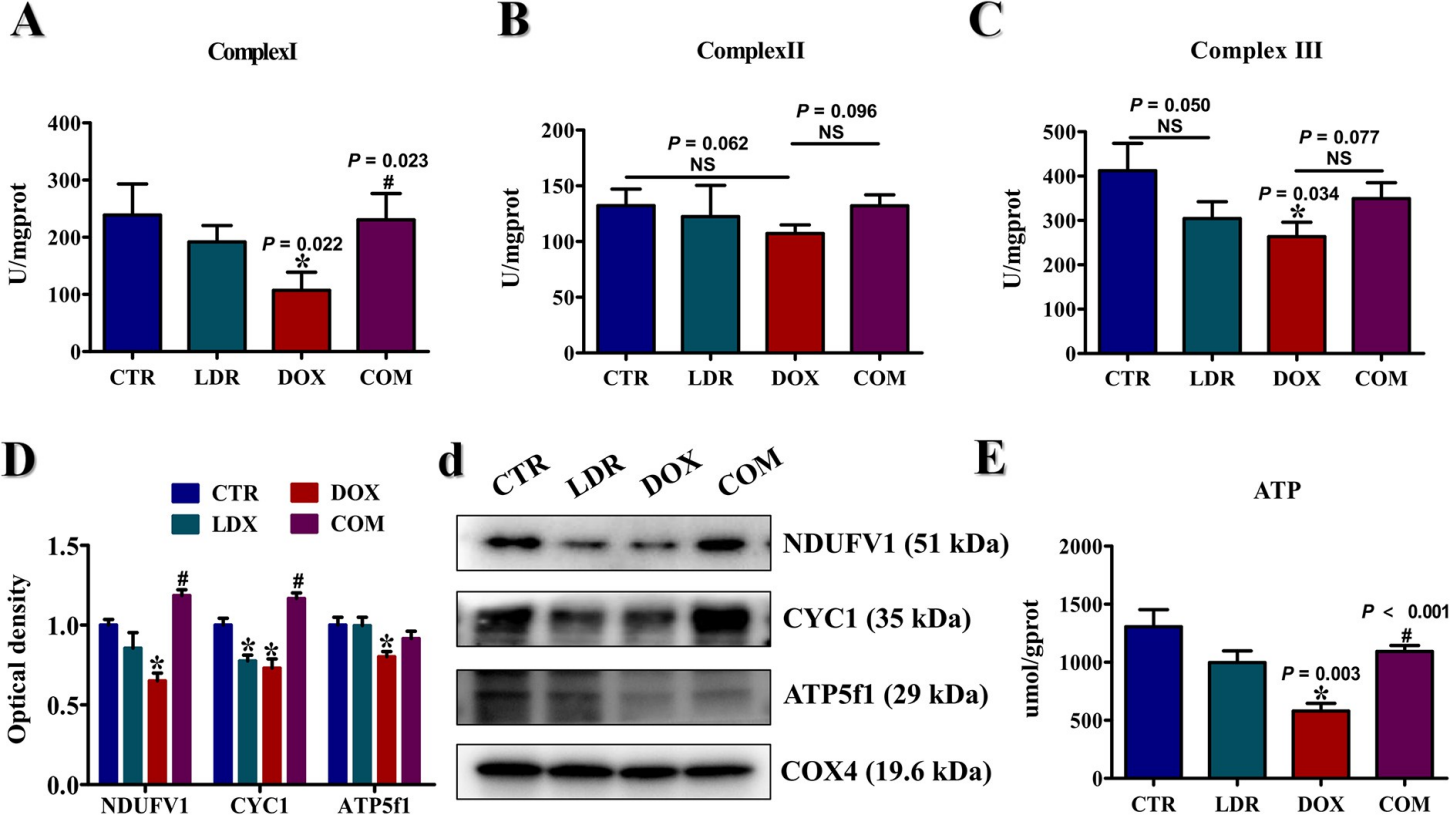
Effects of different interventions on mitochondrial function. The level of complex Ⅰ (A), complex Ⅱ (B), complex Ⅲ (C) in mitochondria. D) The level of NDUFV1, CYC1 and ATP5f1 in mitochondria. E) The content of ATP in kidney tissues. All data were obtained from 3 mice per group. “*” represents the significance difference between treatment group and CTR group (*P*<0.05). “#” represents the significance difference between COM group and DOX group (*P*<0.05). Abbreviation: CTR, control group; LDR, low-dose radiation group; DOX, doxorubicin group; COM, combination group.

The accumulation of intracellular ROS is mainly attributed to the errors of electron transport in the mitochondrial respiratory chain. To investigate the effects of LDR and DOX on mitochondria, the complex Ⅰ, Ⅱ and Ⅲ in mitochondrion were investigated in this study for evaluating the function of mitochondrion (**Fig 6A–6C**). The results showed that the activity of complex Ⅰ in DOX group was significantly less than that in CTR (*P* = 0.022) and COM group (*P* = 0.023). The activity of complex Ⅱ in LDR, DOX and COM group were similar with CTR group. The activity of complex Ⅲ was lower in DOX group than that in CTR group (*P* = 0.034), and there was no significant difference between COM and CTR group (*P* = 0.077).

The expression of mitochondrial complex enzyme subunit markers, such as NDUFV1, CYC-1 and ATP5f1, was further explored to validate the above results **(Fig 6D** and **6d)**. The expression of NDUFV1 in DOX group was significantly lower than that in CTR and COM group (*P*<0.05). The level of CYC1 was significantly decreased in LDR and DOX group (*P*<0.05), and it was up-regulated in COM group compared with CTR group (*P*<0.05). The content of ATP5f1 in DOX group was significantly less than that in CTR group (*P*<0.05). Besides, the level of ATP in kidney tissues was investigated, as shown in **Fig 6E**. Compared with CTR group, the level of ATP was observed to significantly decrease in DOX group (*P* = 0.003). In addition, the ATP content in COM group had significantly higher than that in DOX group (*P*<0.001).

### Supplement

Finally, inspired by previous research [26], the safety of LDR and its effects on liver in mice are also worthy of attention. To verify the effect of LDR on mice liver function, the serum liver function related indexes were tested (**S1A** and **S1B Fig)**. The results showed that there was no significant difference in ALT and AST level between LDR and CTR group. The level of ALT (*P* = 0.0006) and AST (*P* = 0.041) in DOX group were significantly increased compared with CTR group, and ALT level in DOX group was higher than COM group (*P* = 0.01).

H&E staining of liver tissue showed that mild local inflammatory response appeared in LDR group, moderate inflammation was found in the COM group, and liver cell necrosis was observed in DOX group (**S1D Fig)**. Quantitative analysis showed that the liver injury score in DOX group was significantly higher than CTR (*P*<0.001) and COM (*P* = 0.01) group (**S1C Fig)**.

The results of immunofluorescence staining showed that CD45 content in liver tissue was the highest in DOX group, followed by COM, then LDR group, and CTR group (**S1E Fig**).

## Discussion

**Fig 7:**
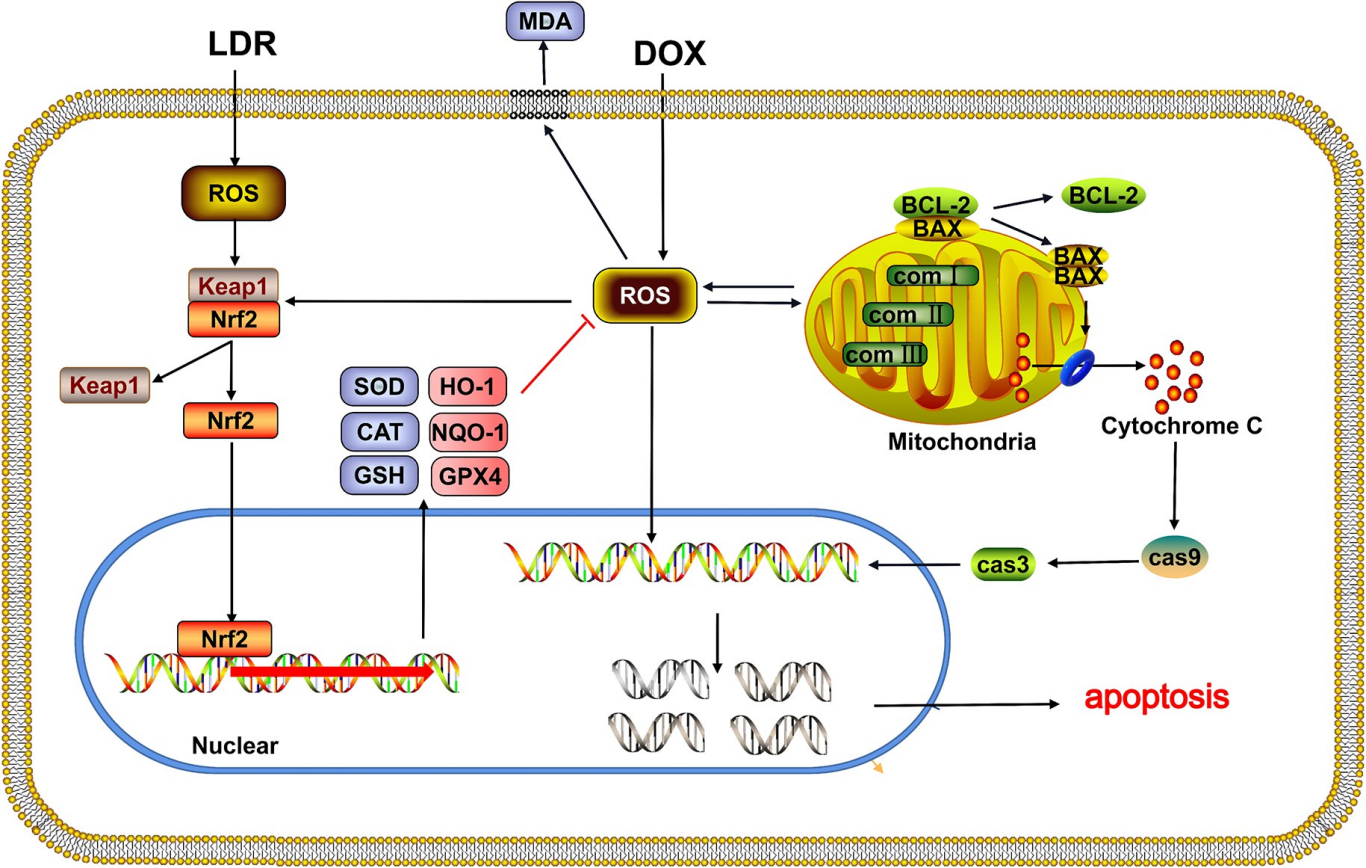
Illustration of low-dose radiation therapy alleviating kidney toxicity induced by DOX. Abbreviations: comⅠ, complexⅠ; comⅡ, complexⅡ; comⅢ, complexⅢ.

As a broad-spectrum anti-tumor medicine, DOX plays an important role in tumor treatment, whereas the application is limited that partly because of severe nephrotoxicity. Previous evidence suggests that the mechanism of kidney cell damage is similar with the heart, which is injured by the oxygen free radicals produced during the metabolism of DOX [27, 28]. Therefore, it is meaningful and necessary to find a safe and effective strategy to reduce oxygen free radical content in kidney and protect DOX-induced nephrotoxicity. LDR is a novel non-invasive approach that shows a protective effect on cardiotoxicity caused by external stimulation [24]. In this study, we verified that renal toxicity induced by DOX could be alleviated by LDR preprocessing. The underlying mechanism might be related to LDR pre-activating the antioxidant pathway, reducing the accumulation of ROS, attenuating mitochondrial damage and kidney cell apoptosis caused by DOX (**Fig 7**).

In order to simulate the application of DOX in gynecological tumors, female BALB/c mice with a gentle personality was selected as animal model. Previous studies have shown that DOX could induce kidney damage in animal models and human biopsy, appeared by the dysfunction of kidney [8, 29, 30]. Therefore, we first evaluated the improvement effect of LDR on nephrotoxicity caused by DOX in mice. Consistent with these previous studies [29, 31, 32], we also found DOX-induced renal toxicity in the present work, as reflected by upregulation of CREA, UREA and UA levels and decreased CO_2_-CP in mice serum. The cytoplasmic vacuolization of renal tubular epithelial cells also demonstrated the presence of renal injury. At meantime, we confirmed that DOX-induced the nephrotoxicity could be alleviated, including the function of kidney and the histological changes through LDR preprocessing. In addition, the safety of LDR on kidney was also confirmed in this experiment.

The apoptosis of kidney cells has been considered as a major factor leading to renal dysfunction and pathological changes [33]. In the present study, we found that DOX significantly upregulated the Bcl-2/Bax-Caspase9 signaling pathway, while the activity of the mitochondria-dependent apoptotic pathways was reduced in the presence of DOX combined LDR. In addition, we also demonstrated that DOX-induced cell apoptosis and kidney damage were attenuated by LDR using more intuitive TUNEL staining. These findings revealed that LDR could attenuate the up-regulation of apoptosis executive proteins induced by DOX.

It is reported that the mitochondria-dependent apoptosis pathway in kidney cells triggered by DOX is mainly due to the DOX-induced oxidative stress, which is caused by the production and accumulation of oxygen reactive substances (ROS) in cells and tissues [34–36]. In cells and tissues, a low level of oxidative stress is necessary for cell mitosis and proliferation, while the moderate and excessive oxidative stress could cause cell growth arrest and death respectively [37]. The study found that the content of ROS in mice kidney were increased after DOX treatment, but the reduction trend of DOX-induced ROS was observed after LDR preprocessing. As a lipid peroxidation product, MDA is produced by free radicals attacking cell membranes. It could reflect the level of lipid peroxidation, thereby indirectly reflecting the degree of oxidative stress damage in tissues and organs [38]. The present study confirmed that the content of DOX-induced MDA in kidney was reduced with LDR preprocessing. Furthermore, the phenomenon that antioxidants activity (SOD, CAT, GSH) was significantly decreased in the DOX group and increased in the COM group also suggested that DOX could induce severe oxidative stress in mice kidneys, while LDR could reduce the accumulation of lipid peroxides induced by DOX.

It is well known that Nrf-2/HO-1/NQO1 signaling pathway is closely related to oxidative stress response [39]. Nrf-2 is a transcription factor that coordinates the basal and stress-inducible activation of a vast array of cytoprotective genes [40]. Normally, Nrf-2 chelates with its endogenous inhibitor Keap-1 in a silent state, in case of oxidative stress Nrf-2 detaches from Keap-1 and trans-locates to the nucleus, where it is involved in regulating the expression of antioxidant genes [41, 42]. HO-1 is an important antioxidant enzyme, which mainly catalyzes the catabolism of heme into ferrous, carbon monoxide and biliverdin [43]. NQO1 can catalyze the reduction of quinone to hydroquinone and prevent oxidative damage to DNA caused by environmental stressors [44]. A large number of studies have shown that this pathway is involved in the antioxidant stress response of heart and vascular diseases and craniocerebral injury, and has defensive and protective effects [45–47]. In this study, the activity of Nrf-2/HO-1/NQO1 signaling pathway was found to be lower in COM group than in DOX group. This phenomenon may be due to the early activation of the antioxidant pathway by LDR, which would result in the rapid scavenging of ROS, making the stimulated signal of Nrf-2/HO-1/NQO1 pathway diminished.

As the main site of cellular energy metabolism, the relationship between mitochondria and ROS is close and complex. Previous studies have found that DOX-induced oxidative stress could not only directly damage cells, but also affect mitochondrial metabolism, leading to the opening of mitochondrial membrane permeability and the disorder of the electron transport system of the respiratory chain [48, 49]. Importantly, ROS is mainly produced in the wrong electron transport process of mitochondrial respiratory chain complex enzymes [50]. Therefore, the effect of LDR on mitochondria of kidney cells was investigated in this experiment, and representative indexes were selected for detection. In line with previous studies, we demonstrated that DOX could induce mitochondrion function injury, especially the electron transport system on which ATP synthesis depends. Although we had no direct evidence to explain the mechanism of LDR on preserving the mitochondrial integrity, LDR preprocessing could reduce mitochondria injury induced by DOX based on the activities of Complex I, II, III, the expression of related subunits of complexes and the level of ATP.

In addition to the protective effect of LDR on kidney damage caused by DOX, it also has similar effects on other organs. In previous studies, our research team found that LDR can protect heart function and brain tissue in DOX-induced damaged mice models [23, 24]. In this study, we also conducted a preliminary study on the effects of LDR on the liver. The preliminary results showed that LDR had no liver damage in mice and could alleviate the liver dysfunction caused by DOX. Furthermore, we found that the expression of CD45 was up-regulated in the liver tissue after the interventions by immunofluorescence staining. As a receptor-linked protein tyrosine phosphatase, CD45 (lymphocyte common antigen) is expressed on all leucocytes, and which plays a crucial role in the function of these cells. Therefore, the up-regulation of CD45 expression may be related to the accumulation of leucocytes caused by inflammation. This finding also suggested that LDR could protect liver function of mice from DOX damage.

In conclusion, this study demonstrated that LDR is a safe and effective method that could reduce DOX-induced nephrotoxicity by protecting mitochondrial function and reducing the production of ROS. Because of these characteristics, LDR might serve as a promising protective measure to prevent tissues damage caused by oxidative stress, and might become a potential candidate to protect cancer patients against chemotherapeutic drug-induced nephrotoxicity. Furthermore, we confirmed that the important role of oxidative stress and mitochondrial damage in DOX-induced nephrotoxicity, providing a reference for finding ways to resist the side effects of DOX.

## Supporting information

S1 Fig Supplementary figure: The effect of low dose radiation on liver injury induced by DOX. (TIF)

S2 Fig The original strip map of western blot. (PPTX)

S1 File Supporting data for the chart. (XLSX)

S2 File Supplementary Table 1: The list of chemical reagents. (DOCX)
