# Supplementary figures and images for: Low-dose radiation ameliorates doxorubicin-induced renal injury via reducing oxidative stress and protecting mitochondrial function
Source: PLoS One. 2025 Feb 11;20(2):e0313649. doi: 10.1371/journal.pone.0313649 (PMC11813107; doi:10.1371/journal.pone.0313649)

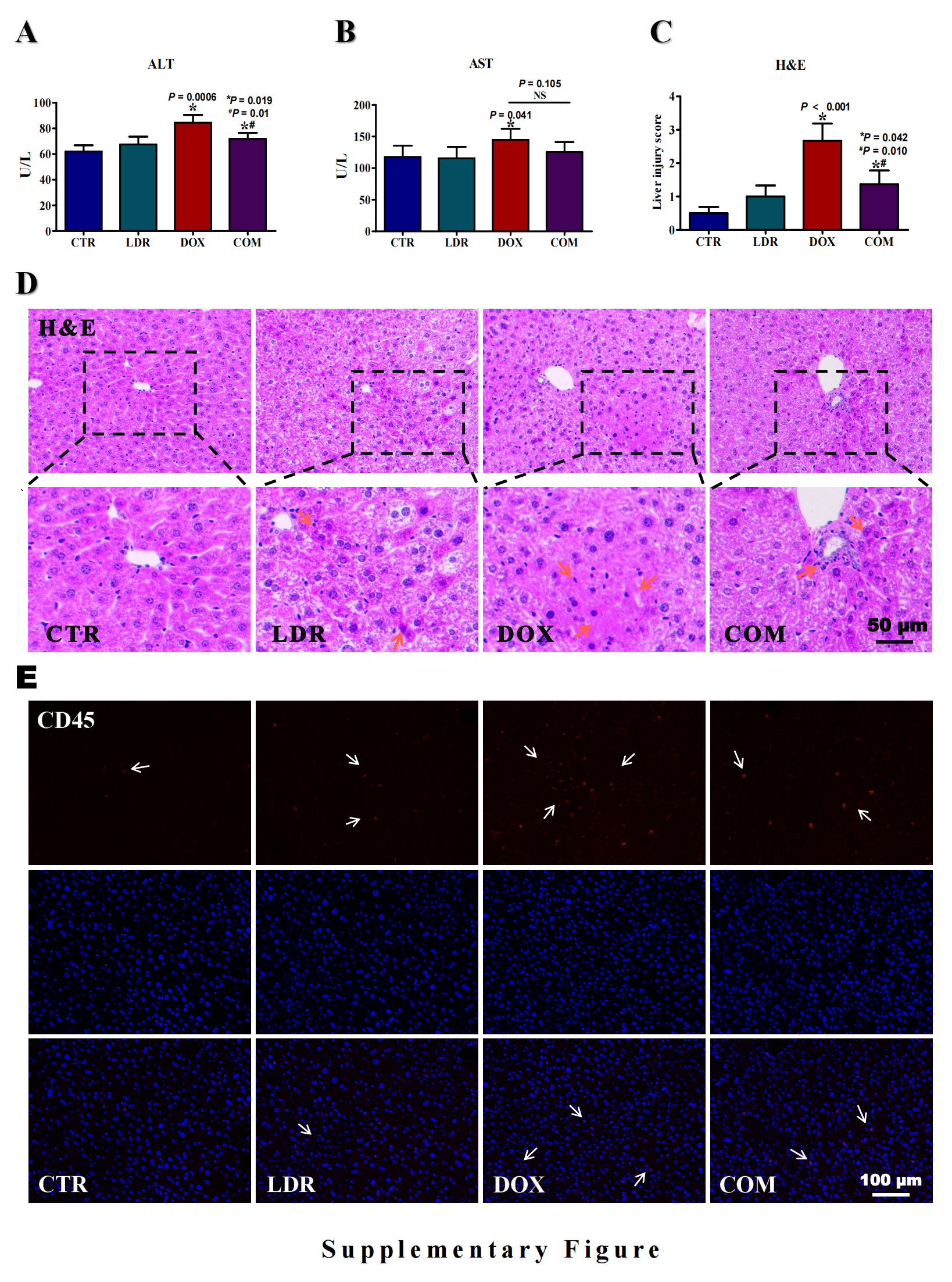

Supplement: S1 Fig — (TIF) [file pone.0313649.s001.tif]
