# Supplementary material for: Low-dose radiation ameliorates doxorubicin-induced renal injury via reducing oxidative stress and protecting mitochondrial function
Source: PLoS One. 2025 Feb 11;20(2):e0313649. doi: 10.1371/journal.pone.0313649 (PMC11813107; doi:10.1371/journal.pone.0313649)
Supplement: S2 Fig — (PPTX) [file pone.0313649.s002.pptx]

## Slide 1
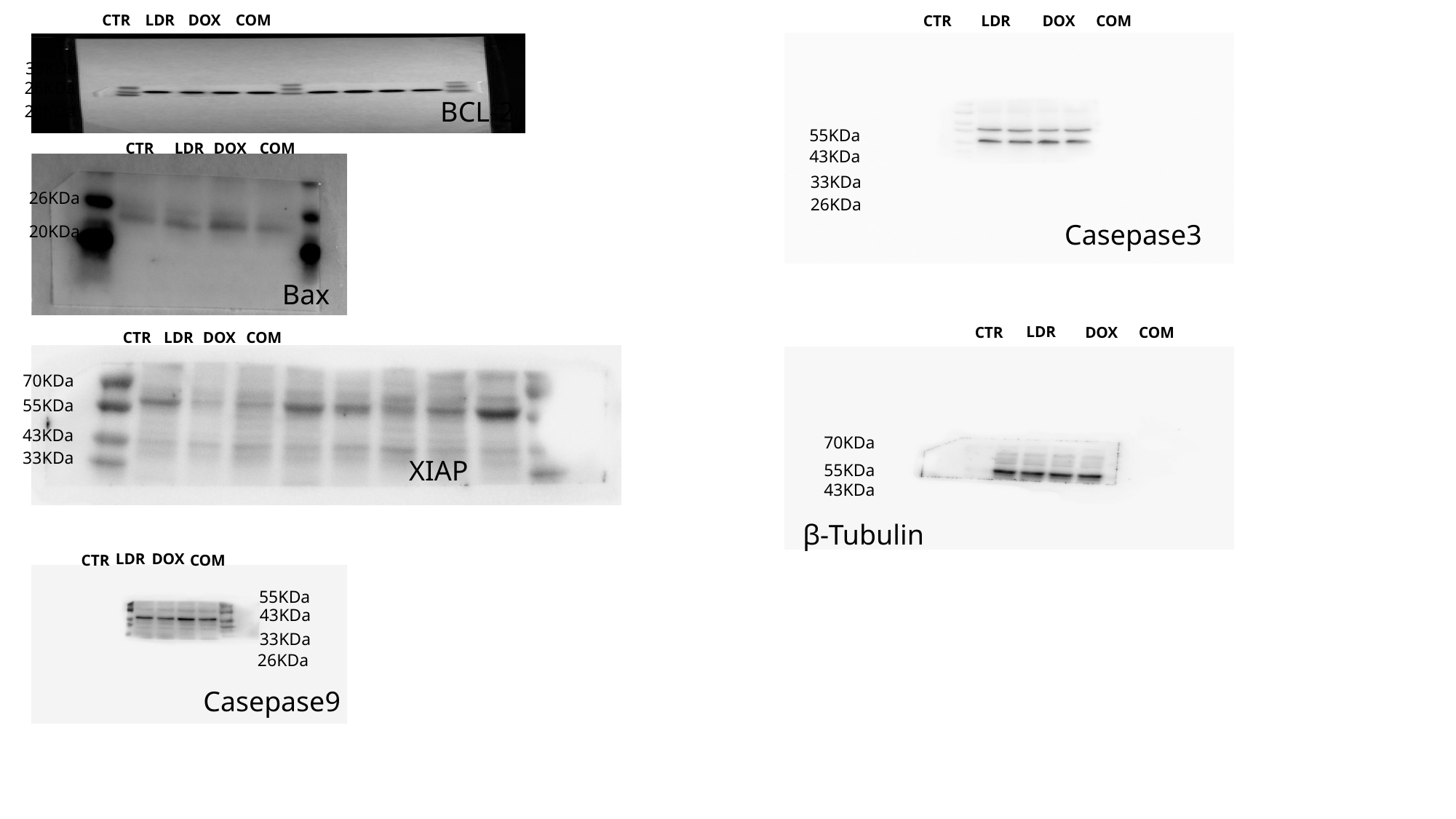

CTR
LDR
DOX
COM
CTR
DOX
LDR
COM
33KDa
26KDa
BCL-2
20KDa
55KDa
CTR
LDR
DOX
COM
43KDa
33KDa
26KDa
26KDa
Casepase3
20KDa
Bax
LDR
CTR
DOX
COM
CTR
DOX
LDR
COM
70KDa
55KDa
43KDa
70KDa
33KDa
XIAP
55KDa
43KDa
β-Tubulin
LDR
DOX
CTR
COM
55KDa
43KDa
33KDa
26KDa
Casepase9

## Slide 2
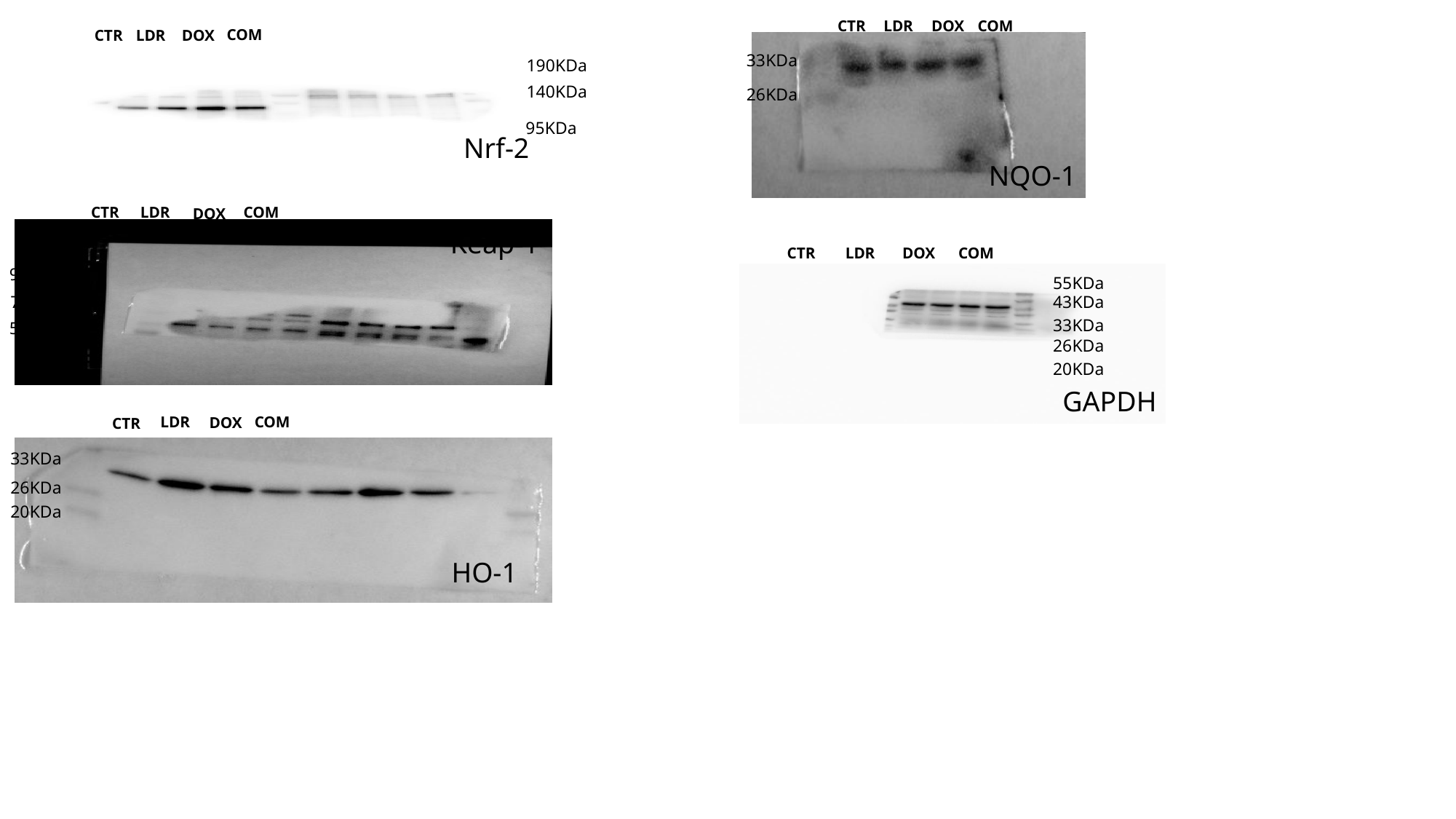

CTR
LDR
DOX
COM
COM
CTR
LDR
DOX
33KDa
190KDa
140KDa
26KDa
95KDa
Nrf-2
NQO-1
LDR
COM
CTR
DOX
Keap-1
CTR
LDR
DOX
COM
95KDa
55KDa
70KDa
43KDa
33KDa
55KDa
26KDa
20KDa
GAPDH
LDR
COM
DOX
CTR
33KDa
26KDa
20KDa
HO-1

## Slide 3
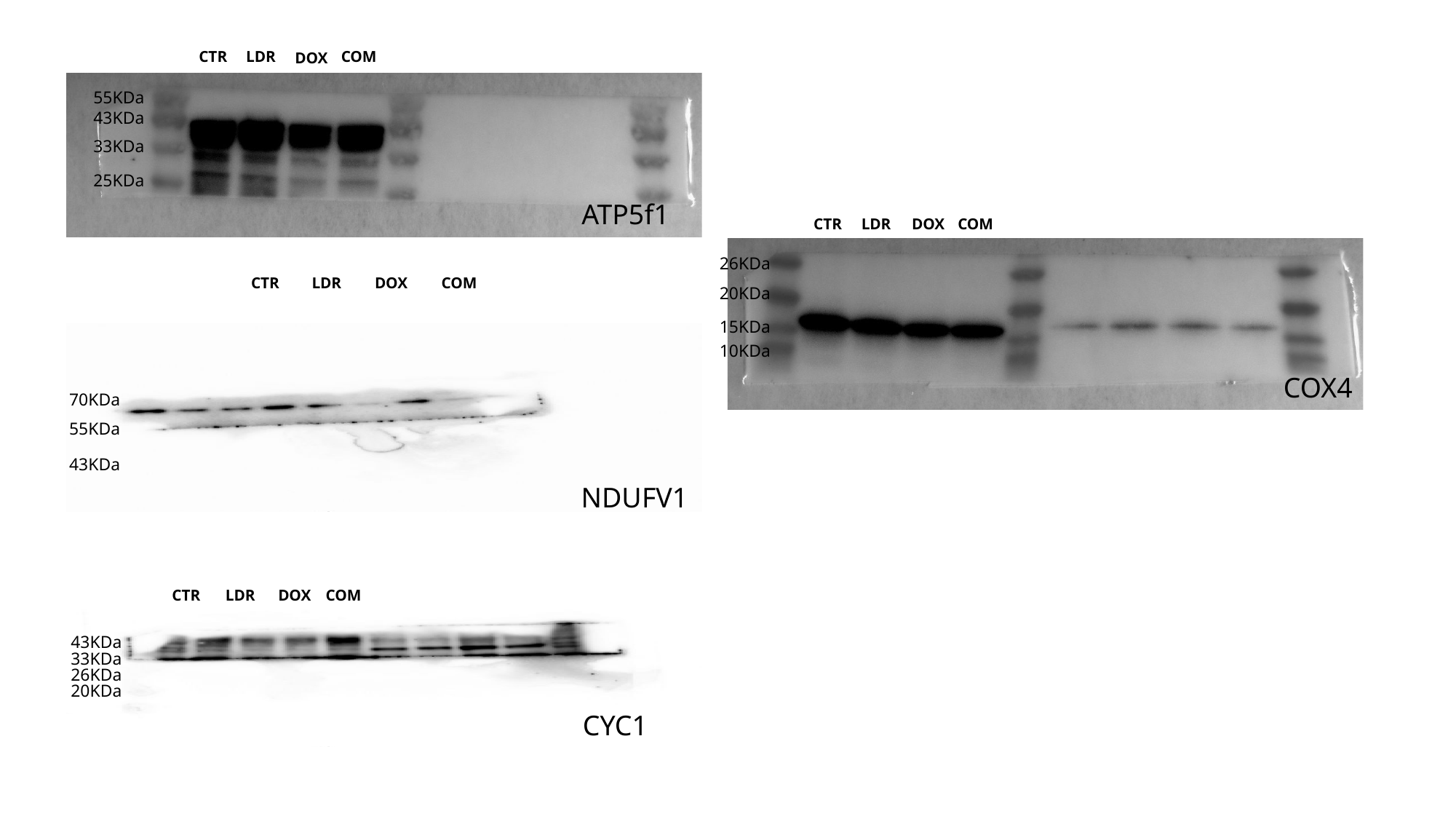

CTR
LDR
COM
DOX
55KDa
43KDa
33KDa
25KDa
ATP5f1
CTR
LDR
DOX
COM
26KDa
CTR
LDR
DOX
COM
20KDa
15KDa
10KDa
COX4
70KDa
55KDa
43KDa
NDUFV1
CTR
LDR
DOX
COM
43KDa
33KDa
26KDa
20KDa
CYC1
