# Supplementary material for: Low-dose radiation ameliorates doxorubicin-induced renal injury via reducing oxidative stress and protecting mitochondrial function
Source: PLoS One. 2025 Feb 11;20(2):e0313649. doi: 10.1371/journal.pone.0313649 (PMC11813107; doi:10.1371/journal.pone.0313649)
Supplement: S2 File — (DOCX) [file pone.0313649.s004.docx]

**Supplementary Table 1. The list of Chemical reagents.**

| **Reagents** | **Cat No.** | **Manufacturers** | **Country** |
| --- | --- | --- | --- |
| Doxorubicin hydrochloride antibody | S17092 | Yuanye Bio-Technology, Ltd | Shanghai, China |
| Anti-B-cell lymphoma-2 antibody | 26593-1-AP | Proteintech Group, Inc. | Chicago, USA |
| Anti-cytochrome C-1 antibody | 10242-1-AP | Proteintech Group, Inc. | Chicago, USA |
| Anti-Caspase9 antibody | 10380-1-AP | Proteintech Group, Inc. | Chicago, USA |
| Anti-cytochrome C oxidase subunit 4 antibody | 11242-1-AP | Proteintech Group, Inc. | Chicago, USA |
| Anti-cluster of differentiation 45 antibody | 80297-1-RR | Proteintech Group, Inc. | Chicago, USA |
| Anti-goat anti-rabbit IgG HRP antibody | SA00001-2 | Proteintech Group, Inc. | Chicago, USA |
| Anti-Bcl2-Associated X protein antibody | AF0120 | Affinity Biosciences, Ltd | Cincinnati, USA |
| Anti-X-linked inhibitor of apoptosis protein antibody | AF6368 | Affinity Biosciences, Ltd | Cincinnati, USA |
| Anti-nuclear factor E2 related factors antibody | AF0639 | Affinity Biosciences, Ltd | Cincinnati, USA |
| Anti-kelch-like ECH-associated protein-1 antibody | AF5266 | Affinity Biosciences, Ltd | Cincinnati, USA |
| Anti-heme oxygenase-1 antibody | AF5393 | Affinity Biosciences, Ltd | Cincinnati, USA |
| anti-nicotinamide adenine phosphate quinone dehydrogenase 1 antibody | DF6437 | Affinity Biosciences, Ltd | Cincinnati, USA |
| anti-subcomplex of the stator of bovine mitochondrial ATP synthase antibody | DF12144 | Affinity Biosciences, Ltd | Cincinnati, USA |
| anti-nicotinamide adenine dinucleotide dehydrogenase (ubiquinone) flavoprotein 1 antibody | DF9668 | Affinity Biosciences, Ltd | Cincinnati, USA |
| anti-β-tubulin antibody | T0023 | Affinity Biosciences, Ltd | Cincinnati, USA |
| anti-glyceraldehyde-3-phosphate dehydrogenase antibody | AF7021 | Affinity Biosciences, Ltd | Cincinnati, USA |
| Terminal deoxynucleotidyl transferase biotin-d UTP nick end labeling (TUNEL) fluorescent assay kit | APT110 | Roche Biotech, Inc | Basel, Switzerland |
| Malondialdehyde assay kit | A003-1-2 | Jiancheng Bioengineering Institute | Nanjing, China |
| Superoxide dismutase assay kit | A001-2-2 | Jiancheng Bioengineering Institute | Nanjing, China |
| Catalase assay kit | A007-1-1 | Jiancheng Bioengineering Institute | Nanjing, China |
| Glutathione assay kit | A005-1-2 | Jiancheng Bioengineering Institute | Nanjing, China |
| Urea nitrogen assay kit | C013-2-1 | Jiancheng Bioengineering Institute | Nanjing, China |
| Creatinine assay kit | C011-2-1 | Jiancheng Bioengineering Institute | Nanjing, China |
| Uric acid assay kit | C012-2-1 | Jiancheng Bioengineering Institute | Nanjing, China |
| Carbon dioxide combining power assay kit | C028-1-1 | Jiancheng Bioengineering Institute | Nanjing, China |
| Adenosine triphosphate assay kit | A095-1-1 | Jiancheng Bioengineering Institute | Nanjing, China |
| Alanine aminotransferase assay kit | C009-2-1 | Jiancheng Bioengineering Institute | Nanjing, China |
| Aspartate aminotransferase assay kit | C010-2-1 | Jiancheng Bioengineering Institute | Nanjing, China |
| Mitochondrial complex Ⅰ assay kit | BC0515 | Solarbio Science & Technology, Ltd | Beijing, China |
| Mitochondrial complex Ⅱ assay kit | BC3235 | Solarbio Science & Technology, Ltd | Beijing, China |
| Mitochondrial complex Ⅲ assay kit | BC3245 | Solarbio Science & Technology, Ltd | Beijing, China |
| Tissue mitochondrial extraction kit | C3606 | Beyotime Biotechnology, Inc | Shanghai, China |
| ROS detection kit | S0033S | Beyotime Biotechnology, Inc | Shanghai, China |
